# Supplementary material for: Do organisms need an impact factor? Citations of key biological resources including model organisms reveal usage patterns and impact
Source: PLoS One. 2025 Aug 13;20(8):e0327344. doi: 10.1371/journal.pone.0327344 (PMC12349098; doi:10.1371/journal.pone.0327344)
Supplement: Attachment — Submitted filename: response to reviewers.pdf [file pone.0327344.s003.pdf]

Dear Editors and Reviewers,

We would like to express our gratitude to the readers of our manuscript who suggested ways to clarify and strengthen the work. We acknowledge that there were some grammatical errors (data plural vs singular etc) that we had made in the original manuscript and have now gone through the manuscript using Grammarly to highlight many of these issues and we fixed those.

We have updated the manuscript and have uploaded the new files, including a version with changes as well as a manuscript version that is clean. We hope that this is now acceptable for publication in PLoS.

As to specific comments, please find them addressed below.

Author affiliations:
All authors who have a SciCrunch affiliation, are clearly listed as having a SciCrunch affiliation. In addition, the conflict of interest statement is included for each SciCrunch affiliated author. One author, Dr. Grethe, is a co-founder of SciCrunch Inc, but holds no position in SciCrunch therefore his affiliation remains only with UCSD, but since he holds significant shares he does have a COI statement. The repositories listed are all not-for-profit so authors affiliated with these repositories are not conflicted.

Retractions:
Thank you for drawing our attention to this key issue. We added a statement in the methods section specifying that: "We did not explicitly exclude retracted papers, but at the time of data collection curators did not find any papers that were retracted."

Please briefly explain what "Citations" versus "Papers" means, in Table 2; i.e., for MMRRC (row 1), 676 citations to MMRRC was made from 511 papers - is this correct

We thank the reviewer for highlighting this key point that might bring confusion about citations of resources vs papers. Indeed it is correct that 511 papers cited 676 mice. A study typically uses ~10 RRIDs, often multiple mice (at least control and experimental groups which are frequently genetically distinct and have distinct RRIDs). We have clarified this point in a few places in the manuscript, including Table 2.

For Figure 2 - please clarify how this data was generated; i.e., is the denominator from the row "Total - animal/antibody was used" in Table 2?

We thank the editor for pointing out another area of potential confusion. There is only a sum and not a denominator for any analysis. We have added some explanatory text into the legend of

table 2, also transcribed below, and we have added the resource type (fish, mouse) into the header row for readability.

"All data are reported as granular citations to a particular mouse or antibody used in a paper (citations) and the number of unique papers that cite the resources (papers). Numbers are counts of resources or individual papers that contain at least one citation of that category, for example 12 antibodies from NHPRR were cited by catalog number in 10 total papers. At the bottom we provide the raw numbers of google scholar searches for these resources with the goal of showing roughly the percentage of papers that were curated for this study vs the number of potential papers that were reviewed. Please note, NHPRR papers curated vastly outnumber google scholar search results because the stock center staff track authors who buy antibodies and follow up with individual authors who may not have mentioned the name of the stock center in their manuscript."

Figure 4
In the case of resources other than MMRRC (thus NXR, AGSC, ZIRC), the authors are asked to comment on whether the variation from linear could be due to the smaller sample sizes.

We have added the following text to the section describing Figure 4 in the text pointing out that the smaller stock centers with fewer papers per year do vary more than the larger stock center.

"Please note that for stock centers other than MMRRC, the variation in average yearly SciScore is larger due to the smaller number of associated papers."

Although perhaps not the focus of this study, it seems appropriate to include specific conclusions regarding the added value of increased use of RRIDs to rigor and reproducibility. This is an early look at the impact of use of RRIDs and yet important trends are clearly emerging and supported by detailed statistical analyses. It is expected that this will be the first in a series of articles on this topic so will become a hallmark of sorts of its own. The use of RRIDs is an important topic that needs to be known about and understood by a larger fraction of the research community so publication is strongly supported. The following are suggestions, all minor, as to ways the manuscript might be improved.

We thank the reviewer in the vote of confidence and we have included new portions of the conclusions/discussion highlighting the role of RRIDs in the quest for improved rigor and reproducibility.

"This change in individual communities of practice centered around the stock center underscores that improvements to rigor and reproducibility practices, especially those that are "about the resources" can indeed be augmented with the help of the stock centers. For investigators, RRIDs primarily serve as a means to ensure that they, or their readers, will easily find the exact research resources used in a study (Bandrowski et al, 2015; Marcus et al, 2016; Bandrowski, 2022), a necessary though not sufficient piece of the reproducibility puzzle. Any help or nudge that authors receive from stock centers and journals simply acts to push more authors to include these bits of unambiguous information about their reagents in their papers."

I think this sentence should be clarified: “Unfortunately, the system is only able to pick up at best about 50% of the citations to mice and antibodies but most of the time the real number is closer to 10% of total due to current citation practices, limiting the effectiveness of this index.” There are other examples that hopefully will be identified and clarified in the detailed editing I encourage.

We have rephrased this sentence to highlight the problems with relying on text mining to find citations to resources. Thank you for pointing out this inconsistency in our language.

“Unfortunately, because the system relies in part on automatic extraction of text, it is only able to “see” about 50% of the citations to mice and antibodies, due in part to licensing restrictions of the content and the citation practices (e.g., nicknaming mice). In most cases, especially with older literature that is licensed with more restrictions and citation practices that rely on nicknames, the percentage of the literature available is closer to 10%, limiting the effectiveness of this index.”

The use of SciScore is key to the analyses but I fear is not well known by most readers so including perhaps more details in the Methods section of what SciScore does and how it does it will both raise awareness of this great tool and also help readers understand its use in this manuscript and the data coming from it.

We thank the reviewer for pointing out that we did not adequately highlight the role of SciScore. We have expanded the methods section describing some of the features of the tool. Also pasted below:

“SciScore is a specialized methods review tool for scientific articles. It employs 55 algorithms to evaluate submitted methods sections against a wide range of rigor criteria known to impact the reproducibility of scientific research. In addition to this, SciScore identifies sentences containing key research resources—such as antibodies, organisms, cell lines, plasmids, and software tools—and assesses how uniquely identifiable these resources are based on the accompanying metadata, e.g., the presence of a catalog number. Using this analysis, SciScore generates a reproducibility score and a detailed report, which includes a rigor adherence table and a key resources table. The adherence of the text to each of the applicable rigor criteria, e.g., inclusion of a statement about investigator blinding, increases the score. The details of the scoring metrics are published (Menke et al, 2022).”

In a few places the authors seem to argue towards a specific conclusion but then fall short of simply stating their preferred outcome. One example, is the first paragraph under Sharing Animals Between Labs, a Word of Caution. Why not end with your point, e.g., “Best practice would argue in favor of including RRIDs for the original source but including a qualifier that

includes the potential for genetic drift." Other examples of stating the value of RRIDs not only for tracking use of resources by stock centers but also to help researchers find them and resulting in increased rigor and reproducibility.

Thank you for asking for boldness. This is important. We have made small changes in several places in the manuscript highlighting the importance of the RRID system and more specifically we have added the sentence suggested about best practices for preventing genetic drift into the discussion section.
