# Supplementary material for: Do organisms need an impact factor? Citations of key biological resources including model organisms reveal usage patterns and impact
Source: PLoS One. 2025 Aug 13;20(8):e0327344. doi: 10.1371/journal.pone.0327344 (PMC12349098; doi:10.1371/journal.pone.0327344)
Supplement: Attachment — Submitted filename: response to reviewers2.pdf [file pone.0327344.s004.pdf]

We would again like to thank Dr. Khan for taking a thorough re-read and pointing out some additional omissions and small errors. We have addressed these in the revised document.

1) Add DSHB to your list of abbreviations, with the full name?
Please see page 1

"DSHB- Developmental Studies Hybridoma Bank (RRID:SCR_013527)"

2) Abstract: Change "From this data, …" to "From these data, …"

"From these data, it was not possible to determine whether outreach to authors or changes to stock center websites drove better citation practices, but findability of research resources and rigor adherence were improved."

3) Define RRID the first time it is used in the text (currently top of p. 5) and consider also doing so in the Abstract as it is such an important part of the article.

Abstract: "The RRID (Research Resource Identifiers) project is filling this gap by working with journals and resource providers to improve citation practices and to track the usage of these key resources."
Also on page 5: "The purpose of RRIDs (Research Resource Identifiers) is primarily to serve investigators to ensure that they, or their readers, will easily find the exact research resources used in a study (Bandrowski et al, 2015; Marcus et al, 2016; Bandrowski, 2022)."

4) Last sentence before Methods section, "university-based resource providers OVER 10 years, spanning the period before and …"

Sentence updated to: "We measured the citation practices of the research community for five university-based resource providers over 10 years spanning the period before and after RRIDs were introduced, and evaluated the ease with which automated routines could identify the organism or reagent used."

5) Heading right at start of Methods should include acronym used repeatedly later as it is the first time used, thus: NIH Office of the Director (OD) funded organism stock centers:

Done: **NIH Office of the Director (OD) funded organism stock centers:**

6) p. 10 under SciScore, define RTI the first time used (you do so later, but…).

Updated sentence to:
“The curator collected the methods section from each paper and obtained the Reproducibility and Transparency Index RTI (Menke et al, 2020) for each paper using the SciScore tool (version 2, https://sciscore.com, RRID:SCR_016251).”

7) Consider reversing the order of the two paragraphs under SciScore so that you first explain what SciScore is and then how you used it.

Done; updated

8) Figure 4 legend appears to have been cut off.
Thank you, it has been recovered.

9) This sentence appears near the top of p. 22: “The main reason that we don’t believe that journals themselves can change resource citation practices is because the NHPRR use case shows that even in places where RRIDs are well accepted, i.e., most journals that enforce RRIDs do so primarily for antibodies, there were relatively few RRIDs for NHPRR between 2014 and 2020.” However, my understanding is that your data suggest that journals CAN have an impact, just that it is smaller than that made possible by the resource centers. Do you mean perhaps “journals alone” instead of “journals themselves”?

“Journals alone” is a better wording for this sentence.

10) On p. 22 add of: “university organism facility for a specified number OF generations, but…”
Sentence updated to:
“Many authors state that the stock came from a stock center and was maintained in the university organism facility over a specified number generations, but in other cases, when labs maintain their own colonies and share resources the provenance of the animal (full name or stock number) can be lost and instead the colonies can be referenced by a nickname.”

The maintenance of the stock in a university facility should be **over** a specified period of time / generations.

11) In the next paragraph on p. 22, “over 10 years before the paper WAS PUBLISHED.”

Updated sentence: “With the help of the author, we tracked down a sale of a single set of founders that was purchased by a nearby lab over 10 years before the paper was published.”

12) p. 23, If we assume that THE Freedman and colleagues…”. In fact, please consider changing to something like the following” If we assume that the Freedman and colleagues

(2017) estimate that 50% of research is not reproducible and that the ""non-findable" resources are the largest culprit (resulting in ~$10.8 billion in waste per year by their estimates) then …

Updated to "If we assume that the Freedman and colleagues (2017) estimate that 50% of research is not reproducible and that the "non-findable" resources are the largest culprit…"

13) Again, I am a bit uncertain as to the conclusions the authors are making as to the role of journals here. As I understand it they believe they can help but not as much as the resource centers, which are the focus of the article. Still, perhaps consider adding at the end of the paragraph at the bottom of p. 23 something like: Although the impact might be expected to be smaller than that provided by the stock centers, journals can also play a meaningful role in increasing the use of RRIDs, increasing rigor and reproducibility and reducing waste.

This sentence fits nicely and was added to the paragraph. We should certainly not let the journals off the hook here.

14) last sentence under Resource Citation Metric, you are missing a space: "antibodies,most"

Added thank you

15) p. 24, are these two sentences intended to be one?: "When considering that there are about 274,000 papers in PubMed Central that referenced mice or antibodies in 2022. One can surmise that even spending 1 minute per citation makes it relatively cost-prohibitive to capture the resource used, as that would entail 2,740,000 minutes or about $4.5M per year in personnel time per year." And consider deleting one of the "per year"s.

Done thank you.
